# Systematic analysis of the transcriptional landscape of melanoma reveals drug-target expression plasticity

**DOI:** 10.1093/bfgp/elad055

**Published:** 2024-01-05

**Authors:** Brad Balderson, Mitchell Fane, Tracey J Harvey, Michael Piper, Aaron Smith, Mikael Bodén

**Affiliations:** School of Chemistry and Molecular Biosciences, University of Queensland, Brisbane, 4072 Queensland, Australia; Fox Chase Cancer Centre, Philadelphia, 19019 Pennsylvania, United States of America; School of Biomedical Sciences, University of Queensland, Brisbane, 4072 Queensland, Australia; School of Biomedical Sciences, University of Queensland, Brisbane, 4072 Queensland, Australia; School of Biomedical Sciences, Queensland University of Technology, Brisbane, 4072 Queensland, Australia; School of Chemistry and Molecular Biosciences, University of Queensland, Brisbane, 4072 Queensland, Australia

**Keywords:** melanoma, metastasis, scRNA-seq, trajectory inference, phenotype switching, drug, cancer

## Abstract

Metastatic melanoma originates from melanocytes of the skin. Melanoma metastasis results in poor treatment prognosis for patients and is associated with epigenetic and transcriptional changes that reflect the developmental program of melanocyte differentiation from neural crest stem cells. Several studies have explored melanoma transcriptional heterogeneity using microarray, bulk and single-cell RNA-sequencing technologies to derive data-driven models of the transcriptional-state change which occurs during melanoma progression. No study has systematically examined how different models of melanoma progression derived from different data types, technologies and biological conditions compare. Here, we perform a cross-sectional study to identify averaging effects of bulk-based studies that mask and distort apparent melanoma transcriptional heterogeneity; we describe new transcriptionally distinct melanoma cell states, identify differential co-expression of genes between studies and examine the effects of predicted drug susceptibilities of different cell states between studies. Importantly, we observe considerable variability in drug-target gene expression between studies, indicating potential transcriptional plasticity of melanoma to down-regulate these drug targets and thereby circumvent treatment. Overall, observed differences in gene co-expression and predicted drug susceptibility between studies suggest bulk-based transcriptional measurements do not reliably gauge heterogeneity and that melanoma transcriptional plasticity is greater than described when studies are considered in isolation.

## INTRODUCTION

Metastatic melanoma is the most aggressive malignancy of the skin, and despite significant advances in treatment modalities in recent years, notably small molecule inhibitors and immunotherapy, long-term prognosis in patients remains poor due to intrinsic or acquired resistance to therapy [1]. Melanoma tumours are amongst the most heterogeneous cancers at both inter- and intra-tumour levels, and this phenotypic plasticity is crucial for both disease progression, dissemination and therapeutic resistance [1–3]. A propensity for phenotype switching has been recognised in melanoma tumours, whereby cells are able to transition between proliferative’ and invasive’ states, with cells undergoing transcriptional reprogramming governed by distinct transcriptional regulators, most notably the MITF melanocytic master regulator [4–6].

Numerous signalling pathways, changes in the tumour microenvironment and therapeutic stress influence the transitions between alternative transcriptional states, with myriad alternative phenotypes likely to exist within tumours [1, 7]. Recent analysis of low-passage melanoma cell line bulk transcriptomes by Tsoi *et al*. (2018) led to the suggestion that melanomas de-differentiate through four distinct states; melanocytic (proliferative) state, to transitory state, to neural crest-like, before finally adopting an undifferentiated stem cell (invasive) state [8]. This trajectory model corroborated the proliferative-invasive model, showing a gradual reduction in expression of the melanoma master transcription factor MITF, and a corresponding increase in expression of genes prognostic for treatment resistance such as the receptor tyrosine kinase AXL and the growth factor receptor EGFR [1, 9]. Further, based upon classifying melanoma cell lines treated with a battery of drugs in the LINCS1000 database [10], Tsoi and others were able to identify ferroptosis inducers as a novel class of drugs that target the de-differentiated/invasive melanoma cell states.

Bulk expression-based studies that examine melanoma heterogeneity using transcriptional profiling are powerful [8, 11], and have significantly advanced our understanding of diverse cell states in melanoma. However, the bulk expression measurements, potentially miss additional heterogeneity that may occur within tumours profiled using state-of-the-art single-cell methods. Rambow *et al*. (2018) describe dynamic changes in PDX tumours derived from seeded clonal melanoma cells in response to RAF/MEK inhibition [7], identifying five transcriptionally distinct melanoma cell states, two of which were induced during the drug treatment. These states were named pigmented, proliferative, starved-like melanoma cells (SMCs), neural crest stem cell-like (NCSC) and invasive melanoma. Importantly, the NCSC and invasive melanoma cell states, which developed resistance to MAPKi treatment, were driven by the nuclear receptor RXRGamma and could be effectively targeted with small molecule antagonists to attenuate the resistant states. The utilization of mouse models by Rambow and others raises a question of whether the observed cell-state transition and the genes involved therein translate to the context of human tumours.

Similarly, Tirosh *et al*. (2016), performed single-cell RNA-seq directly from 19 human patient tumours [9] Melanoma heterogeneity was examined using a supervised approach in this study, primarily comparing MITF high proliferative melanoma cells and AXL high invasive melanoma cells [9, 11]. Possibly owing to the lack of methods to correct for unwanted variation in single-cell RNA-seq at the time, an attempt to analyse these data in an unsupervised manner without prior assumptions of the transcriptional heterogeneity was not performed. Given this data are the most clinically relevant, containing single-cell resolution profiles from individual patient tumours, it presents a great opportunity for re-examination in an unsupervised manner to test for additional melanoma heterogeneity.

Rambow and Goding (2019) have argued for a unifying nomenclature of melanoma states based on the heterogeneity identified over the preceding 15 years through a combination of higher resolution transcriptomics and improved experimental design [12] Ordered from differentiated/proliferative to invasive, the nomenclature proposed is: hyper-differentiated, melanocytic, intermediate, starved, undifferentiated and neural-crest-stem-cell like. Given the clinical importance of defining melanoma heterogeneity and the historical success of this approach to identify new drugs that target particular cell states [8, 11, 12], here we employ the Tirosh scRNA-seq dataset to derive a trajectory describing melanoma de-differentiation from human patient tumours, and systematically compare this to the studies by Hoek and Goding [11], Tsoi [8], The Cancer Genome Atlas (TCGA) [13] and Rambow [7]. The aim of this comparison is to examine whether transcriptional states which are assumed to be equivalent between studies actually are, and to identify the differences between studies to yield insights regarding limitations in technology for defining cell states or biological differences that could indicate greater melanoma heterogeneity than would be apparent when each dataset is considered in isolation. This is largely motivated by similar cell atlas studies, which collate scRNA-seq datasets for a particular biological tissue to create a consensus with regards to cellular diversity [14, 15].

Using the compiled data representing melanoma transcriptional heterogeneity across a variety of biological conditions, we then use drug perturbation profiles from the LINCS1000 database to predict drug sensitivity of the identified melanoma states within each study [10, 16]. This analysis suggests several classes of drugs such as glucocorticoid receptor (NR3C1) agonists, AXL inhibitors, PARP1 inhibitors and HDAC inhibitors as the most consistently expressed candidate drug-treatments in invasive melanoma across all studies examined.

## RESULTS

### Trajectory analysis infers branching of melanoma in vivo de-differentiation

**Figure 1:**
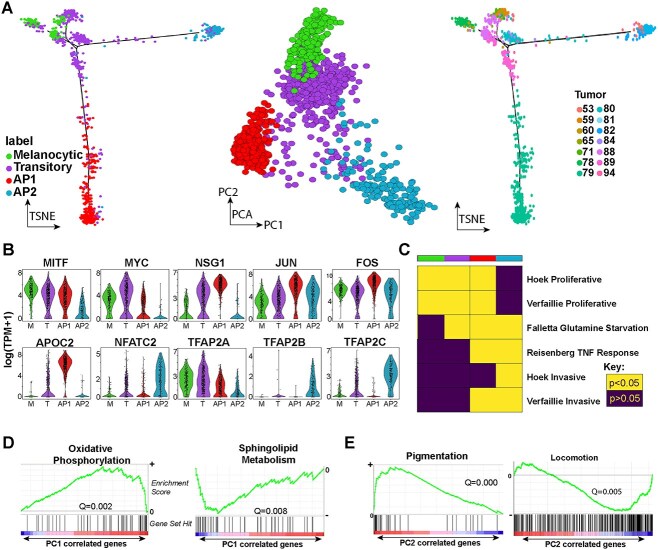
Re-examination of Tirosh single-cell RNA-seq data reveals a trajectory with two states of melanoma transition. (**A**) Left: tSNE of the trajectory derived using Monocle and coloured according to the cell states derived from hierarchical clustering; Melanocytic (green), Transitory (purple), AP1 invasive state (red) and AP2 invasive state (blue). (Middle) The same cell states visualised in PCA space derived from relative gene expression. (Right) The Monocle tSNE trajectory coloured according to patient tumour sample. (**B**) Violin plots of gene expression (log2(TPM+1)) of notable genes in melanoma metastasis biology in each cell state. The cell state legend is shared with (A). (**C**) Enrichment of cell state prognostic genes in various melanoma gene sets derived from independent data; each column is a cell state, and each row is an independent melanoma gene set. Yellow indicates statistically significant (Fisher’s exact test) enrichment for the cell state marker genes in the respective melanoma gene set. (**D**) GSEA results from searching for gene enrichment in the Molecular Signature Database for genes ranked according to correlation with principal component one (PC 1; see A, middle). Significant gene sets of oxidative phosphorylation, and sphingolipid metabolism are shown with significant positive and negative enrichment, respectively. (**E**) The same as (D), except for genes correlated with PC 2, indicating positive and negative enrichment for pigmentation and locomotion, respectively.

To compare cell states across studies, we first sought to derive a model of melanoma transcriptional heterogeneity using data with high clinical relevance to provide a baseline for comparison. In contrast to the original analyses of these data which compared MITF high proliferative melanoma cells against AXL high invasive cells, the 1252 melanoma scRNA-seq profiles from 19 patients generated by Tirosh *et al*. (2016) was analysed in an unsupervised manner, without prior assumption of the apparent heterogeneity. Monocle [17], hierarchical clustering and principal components analysis (PCA) was utilized to derive a single trajectory by two independent approaches (see Methods). This revealed four melanoma states, which we further characterised by calling differential gene expression using Wilcoxon Rank-Sum tests with a one-versus-rest mode of comparison (Figure 1A, Table S1). Further genes can be queried for their expression in these states via http://bioinf.scmb.uq.edu.au:81/SingleCellUniverse/melanoma/.

The unsupervised analysis revealed a more nuanced reflection of melanoma de-differentiation than a single trend of MITF high to MITF low melanoma cells. In-line with previous studies, we identified a MITF high state, which we refer to as Melanocytic, due to the expression of melanocyte markers such as PMEL, TYR and TFAP2A. We also identified an intermediate cluster of cells with an intermediate level of MITF expression and expression of genes involved in stemness such as MYC and NFATC2; we refer to this state as Transitory (Figure 1B, Table S1). However, in contrast to the melanoma rheostat model, from the transitory state, we observed two distinct branches of melanoma transition, with one branch showing complete reduction in MITF and the other an intermediate reduction (Figure 1B, Table S1). Between these two branches, we identified several points of difference, in particular along one branch we observed upregulation of FOS and JUN (the AP1 transcription factor complex [6]). Along the other branch, we observed upregulation of the NFI transcription factors, and less well-studied transcription factors in melanoma biology such as TFAP2B and TFAP2C (AP2 transcription factors [18]). We hence refer to these two distinct melanoma states as the AP1 and AP2 states, respectively.

#### Gene enrichment analyses suggests two distinct invasive states

Our analysis of the Tirosh scRNA-seq data revealed divergent melanoma transcriptional phenotypes that correlate with low MITF expression. To relate the melanoma states identified to previously published data, we selected the top 500 upregulated genes in each state from the differential expression (DE) gene lists and tested for enrichment of these genes in previously characterised gene sets involved in melanoma migration (Figure 1C, Table S2, Supplementary Fig. 1). These gene sets were originally compiled by Falletta *et al*. (2017), and included proliferative and invasive melanoma signatures [6, 11], and melanoma signatures from glutamine starvation and treatment with TNF-alpha, which has been shown to induce melanoma migration [19, 20].

These enrichment analyses showed the AP1, AP2, Transitory and Melanocytic marker genes were all enriched in at least one of the melanoma gene sets. The most salient observations included AP1 and AP2 gene enrichment in melanoma TNF response, AP1 gene enrichment in the Verfaillie invasive gene set and AP2 enrichment in the Hoek invasive gene set. Upregulated genes in the AP1 and AP2 states were thus consistently enriched in gene signatures that are associated with melanoma invasion and migratory capacity.

We also leveraged gene set enrichment analysis (GSEA) on gene sets in the Molecular Signature Database to probe for gene sets that were significantly (anti)correlated with the PCs that separated these cells [21, 22]. PC 1, which separates the AP1 and AP2 states, was positively correlated with genes involved in oxidative phosphorylation and negatively correlated with genes involved in sphingolipid metabolism (Figure 1D). This suggests the main separation between the AP1 and AP2 states is due to expression of metabolically distinct genes.

PC 2, in line with the trajectory analysis, showed positive correlation with pigmentation genes and negative correlation with locomotion genes (Figure 1E). Thus, the PC 2 axis is in-line with the melanoma rheostat model, ordering melanoma scRNA-seq profiles from melanocyte-like to invasive. Taken together, the enrichment analyses thus point toward the AP1 and AP2 states representing two distinct invasive states of melanoma induced by de-differentiation but separated by distinct metabolic gene signatures.

### Comparison with Tsoi bulk trajectory suggests averaging effect masks melanoma heterogeneity

**Figure 2:**
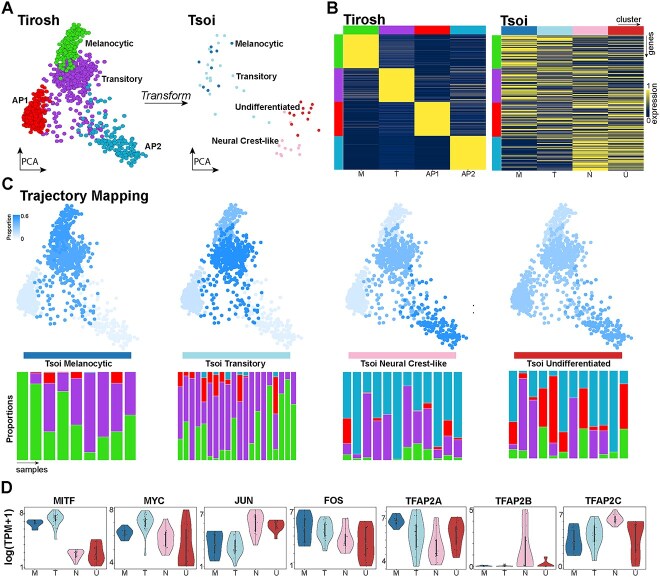
Deconvolution of melanoma trajectory by Tsoi reveals bulk RNA-sequencing masked invasive heterogeneity. (**A**) (Left) is the Tirosh trajectory, (right) is the Tsoi trajectory transformed into the same PCA space. (**B**) (Left) is a heatmap of average gene expression for marker genes of the Tirosh states, with rows being the genes coloured according to the Tirosh state they demarcate, and columns referring to the Tirosh states. (**B** (Right) shows an equivalent heatmap of the Tirosh marker genes except the columns correspond to the Tsoi clusters. (**C**) The Tirosh trajectory with each of the single-cell states coloured according to the average proportion of that state in the respective Tsoi bulk states; for each of the Tsoi melanocytic, transitory, neural crest-like and undifferentiated clusters, respectively. Below each trajectory is a bar plot; with each bar corresponding to a particular Tsoi bulk sample. The bars are coloured according to the estimated proportions of the Tirosh single-cell states in the respective bulk sample; with bulk samples grouped according to the respective Tsoi cluster. (**D**) Violin plots of notable genes in the Tirosh trajectory in each of the Tsoi clusters.

Why were the two novel invasive states not identified in the original trajectory determined by Tsoi and colleagues [8]? We hypothesised that the bulk gene expression measurements may have obscured the two distinct melanoma invasive states. To test this, we first reproduced the Tsoi trajectory using their publicly available data (Figure 2A, D). We then applied the PCA transformation learnt from the Tirosh single-cell data to the Tsoi bulk expression data to observe the bulk trajectory in the same gene expression space. This revealed that the Tirosh trajectory space separated the Tsoi bulk states into proliferative and invasive cell states, showing overlapping biological variability driving the clustering of both datasets (Figure 2A). We then visualised highly and specifically expressed marker genes for each of the Tirosh states in the Tsoi clusters, showing the bulk samples included mixtures of marker gene expression from different single-cell clusters (Figure 2B). Estimating the proportion of the different Tirosh single-cell states in the Tsoi bulk expression profiles using the CIBERSORT [23] method revealed samples from the Tsoi undifferentiated cluster represent complex mixtures of the Transitory, AP1 and AP2 cell types (Figure 2C). Hence the two invasive states were not detected in the Tsoi bulk trajectory analysis due to the limitations of bulk measurements averaging the cellular heterogeneity.

### Mapping melanoma states implies presence in samples from TCGA

**Figure 3:**
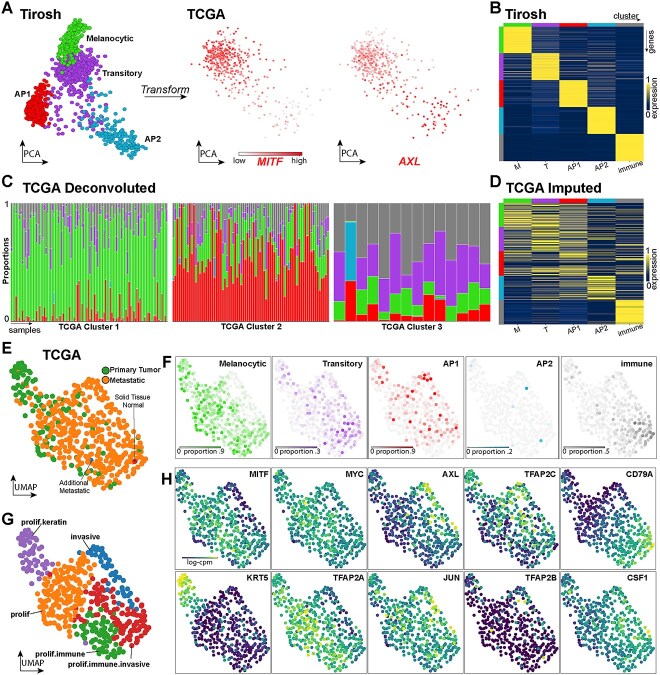
Analysis of TCGA melanoma bulk RNA-seq reveals equivalent gene expression trends in melanoma metastasis. (**A**, left) The Tirosh melanoma cell states trajectory. (**A**, right) 469 bulk melanoma tissue samples from TCGA transformed into the same PCA space as the Tirosh trajectory and coloured according to MITF and AXL levels of gene expression. (**B**) Heatmap of Tirosh marker genes selected to differentiate the melanoma cell states as well as tumour infiltrating immune cells; the rows indicate genes, and the columns cell states. Rows are coloured according to the cluster they demarcate. Cell state colours are the same as indicated in (A) left, with the addition of immune cells indicated by grey. (**C**) Bar plots showing the estimated proportions of the different cell states described in the TCGA samples. Each bar represents a single sample, and the area within the bar is coloured according to the proportion of the different cell states estimated within the respective sample. Samples are grouped into clusters of samples by different proportions estimated in those samples. Cluster 1 shows a representative 100/379 samples for visual clarity. (**D**) Heatmap with the same features described from (B), except showing the estimated expression of the different melanoma cell states derived solely from the TCGA bulk samples based on the estimated cell state proportions in each sample (see the text). (**E**) The TCGA samples clustered in Uniform Manifold Approximation (UMAP) space and coloured according to cancer status. (**F**) TCGA UMAP with samples coloured by the estimated proportions of the different Tirosh states (shown as bar plots in C). (**G**) Labelling of different TCGA clusters based upon the deconvolution results in (F), and the complementary expression of marker genes for the different states in (H).

We next sought to infer the presence of our melanoma states in tissue samples derived directly from patient tumours in TCGA [13]. We downloaded a total of 469 bulk RNA-seq samples from the Skin Cutaneous Melanoma (SKCM) project and normalised the data for comparison with the Tirosh single-cell data (see Methods). Transforming the TCGA bulk data into the same PCA space as the Tirosh trajectory separated the bulk samples in a linear proliferative to invasive trajectory, as indicated by visualizing MITF and AXL expression levels (Figure 3A). Hence, the derived PCA space is reflective of the MITF rheostat model when applied to bulk RNA-seq data, but reveals greater heterogeneity in the invasive cells when used to display the Tirosh scRNA-seq data.

The proportion of the different melanoma states in the TCGA bulk RNA samples was estimated using marker genes that distinguish the melanoma cell states and also immune cells, present as an effect of the samples being derived from in vivo tumours (see Methods, Figure 3B, Table S2). Samples clustered based on their cell state proportions using a method that dynamically learns the number of clusters revealed three distinct clusters of TCGA samples (see Methods, Figure 3C). Cluster 1 samples were primarily composed of Melanocytic cells, cluster 2 of AP1 cells, and cluster 3 of Transitory and immune cells. Only three samples had estimated proportions of AP2 cells above 0.1, one of which can be clearly observed in the third TCGA cluster as the second sample (Figure 3C). The proportion estimates were then used to impute the cell state specific gene expression from the TCGA bulk samples, yielding similar gene expression profiles to those we observed in the Tirosh data (Figure 3B, D). Overall, this analysis confirms the cell states identified in the Tirosh scRNA-seq data are present in the TCGA bulk samples.

Further, based upon the expression of marker genes for the states identified in the Tirosh data (Figure 3E-H), we labelled the TCGA samples based on relative invasiveness and immune content. This lead to the identification of five clusters reflecting different cell type mixtures; proliferative/keratin (prolif.keratin, primary melanoma), proliferative (prolif, metastatic melanoma with proliferative signature, MITF high), invasive (AXL, JUN, TFAP2B, TFAP2C high), proliferative/immune (prolif.immune, metastatic melanoma with proliferative and immune signatures) and, finally, proliferative/immune/invasive (metastatic melanoma samples with proliferative, immune and invasive gene signatures expressed). Notably, these cluster annotations correlated strongly with the estimated proportions of the different melanoma states and immune content proportions estimated using the Tirosh single-cell data as a reference (Figure 3F).

### Integrative analysis with single-cell RNA-seq from Rambow *et al*. finds comparable cell states but differentially co-expressed genes

**Figure 4:**
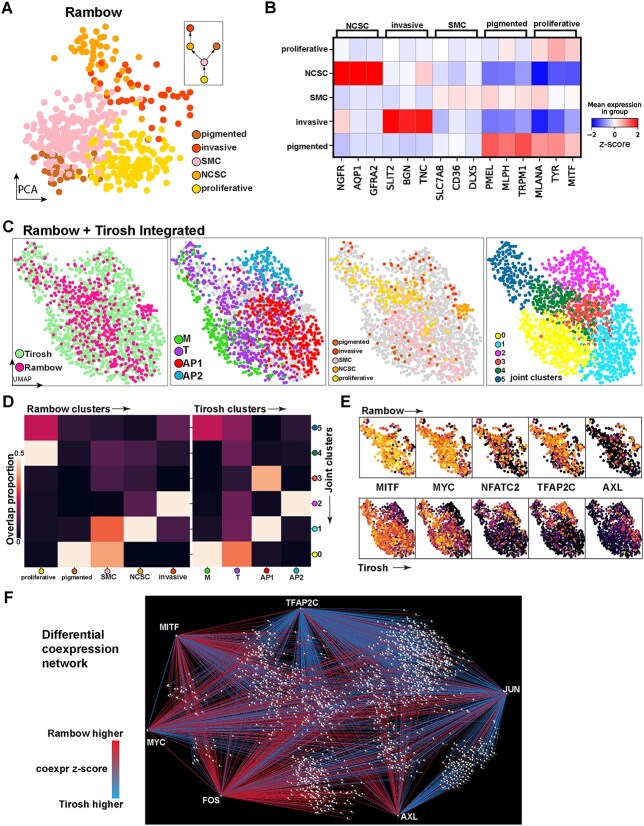
Integrative analysis of clonal single-cell RNA-seq trajectory from Rambow reveals equivalent cell states and differential coexpressed genes between studies. (**A**) Single-cell RNA-seq profiles from Rambow coloured by cell state (annotations provided by authors). (**B**) Heatmap shows marker genes for the different cell states by Rambow. (**C**) UMAP of Rambow and Tirosh single-cell data integrated into a common dimensionality reduction while controlling for study effect. The UMAP is repeated four times, coloured by; dataset, Tirosh states, Rambow states, and clusters from analysing the data jointly. (**D**) Heatmap showing the proportion of cells from each cell state appearing in a joint cluster. States appearing in the same joint cluster across studies indicate equivalent cell states. (**E**) Expression of melanoma biology genes in the joint space, with the Rambow cells above and the Tirosh cells below. Several genes appear in equivalent regions of space, but others (NFATC2, TFAP2C) appear more broadly expressed in the Rambow data. (**F**) Network showing significant results from performing differential coexpression analysis between the Rambow and Tirosh data. Each node is a gene. Red edges indicate increased coexpression in the Rambow data, while blue edges indicate reduced coexpression.

We then sought to reveal which of the four states in the Tirosh scRNA-seq data are equivalent to the five melanoma states identified by Rambow and colleagues, allowing for a cross-study comparison of differences between states (Figure 4A–B). To achieve this, we first integrated the two studies into a common UMAP space (see Methods), effectively grouping cells across studies by maximum similarity while controlling for study-specific variation (Figure 4C). We then clustered the cells from each study within this space to obtain six joint clusters (Figure 4C, right). For each joint cluster, the proportion of cells within the cluster belonging to the Tirosh and Rambow cell states were calculated (Figure 4D). The proliferative, pigmented and SMC melanoma cells in the Rambow data clustered with the melanocytic and transitory states identified in the Tirosh data in joint clusters 0, 4 and 5 (Figure 4D). The Rambow NCSC and the Tirosh AP1 state were clustered in joint cluster 1 (Figure 4D). Joint cluster 2 was primarily composed of the Rambow invasive state with the Tirosh AP2 state. This integrative analysis with the Rambow single-cell data thus supports earlier observations of the probable invasiveness of the states observed in the Tirosh data, with the two invasive states in Rambow clearly mapping to the two invasive states identified in the Tirosh data.

Importantly, the correlation of TFAP2C with melanoma invasive signatures was not observed by Rambow, et al, leading us to examine the expression of key marker genes from the Tirosh states in the Rambow data (Figure 4E). We observed several genes with equivalent gene expression trends between the two studies; MITF, MYC and AXL were high in the proliferative, transitory and invasive states, respectively (Figure 4E). However, NFATC2 and TFAP2C, key markers of the AP2 invasive state identified in the Tirosh data, were broadly expressed throughout the Rambow melanoma cells. This was confirmed statistically using differential co-expression analysis, whereby TFAP2C was positively correlated with MITF in the Rambow data, while TFAP2C and many other AP2 state genes in the Tirosh data were anti-correlated with MITF in the Rambow data (Figure 4F).

While the mechanism underlying the difference in gene co-expression remains to be determined, the biological differences between the two datasets, such that the Rambow data represent clonal cells in active transition due to RAF/MEK inhibitor treatment, may suggest a key role of TFAP2C in this transition process that is, in part, independent of MITF.

### Meta-analysis summarises comparable states across studies

**Figure 5:**
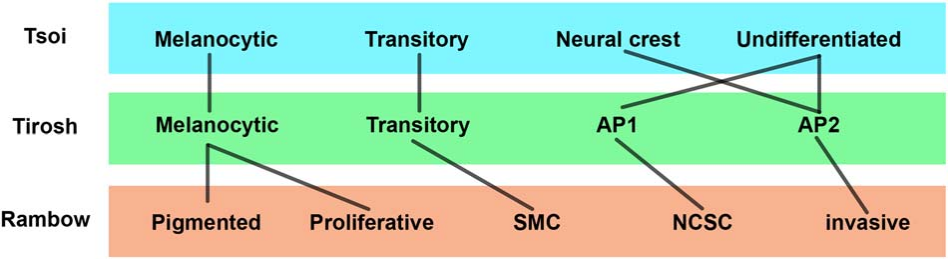
Trajectory comparison schematic. Each row refers to a different dataset. Melanoma cell states identified for the respective study are indicated within the coloured bars (Tsoi, blue; Tirosh, green; Rambow; orange). Black bars between cell types indicate comparable cell states across studies.

Based on our cross-study comparison of melanoma datasets, we summarize equivalent transcriptional melanoma states between studies in Figure 5. The Tirosh AP2, Tsoi neural-crest and Rambow invasive states are transcriptionally equivalent (Figure 5). The Tirosh AP1 state is comparable with the Rambow NCSC state, while the Tsoi undifferentiated state is a mixture of Tirosh AP1 and AP2 cells.

### LINCS L1000-based drug-susceptibility prediction across studies by BeyondCell indicates poor cross-study overlap at the individual drug level

**Figure 6:**
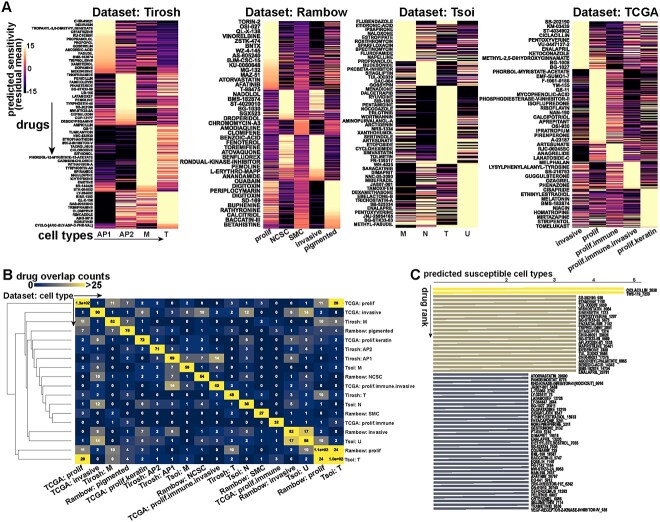
BeyondCell drug susceptibility analysis across studies indicates poor overlap in predicted drug susceptibility. (**A**) Heatmaps from performing BeyondCell drug susceptibility analysis on each dataset independently. Each row in the heatmap is a drug, and each column is a melanoma cell state identified in the respective study. Bright values indicate greater predicted drug sensitivity, measured by the BeyondCell residual mean (see Methods). (**B**) Symmetric heatmap with cell states listed on both the rows and the columns, with numbers indicating the overlap in the number of drugs both states are predicted to be susceptible to. (**C**) Bar plot ranking drugs by the number of cell states they are predicted to target, regardless of the study.

Melanoma cell state drug susceptibilities were predicted within each study using BeyondCell [16] (Figure 6A, Table S3; see Methods). The overlap of drugs predicted to target each study revealed poor consistency of predicted drugs between studies, with a maximum of three or less drugs found in common between different combinations of study and cell states (Figure 6B). Exceptions are notable; with the Rambow proliferative state and the Tsoi transitory state sharing 24 predicted drug sensitivities, the TCGA proliferative and Tsoi transitory sharing 28 drugs, the TCGA invasive and the Tsoi undifferentiated sharing 14 drugs, and the Tirosh AP1 and Rambow NCSC sharing seven drugs. The study/cell state combinations with the highest drug susceptibility overlaps commensurate our previous analyses regarding equivalent states identified across studies.

Ranking drugs by the number of cell states targeted (Figure 6C, Table S3) revealed several promising but many more unlikely drugs (based on mechanism of action, MOA) targeted the largest number of melanoma states; for example, Ciclacillan [24] (beta-lactam antibiotic), TWS-119 [25] (glycogen kinase 3-beta inhibitor), SB-202190 [26] (p38 MAPK inhibitor), Wiskostatin [27] (N-WASP inhibitor), Ethamivan [28] (respiratory stimulant) and Enalapril [29] (ACE inhibitor). While TWS-119 and SB-202190 may have some efficacy, given their MOA has been previously shown to affect melanoma [30, 31], the other top-ranking drugs have unknown efficacy. The number of unlikely drugs based on MOA prompted us to examine drug classes that may consistently target the invasive melanoma cell states, rather than individual compounds.

### Analysis of the drug perturbation landscape reveals plausible drug classes for targeting invasive melanoma

**Figure 7:**
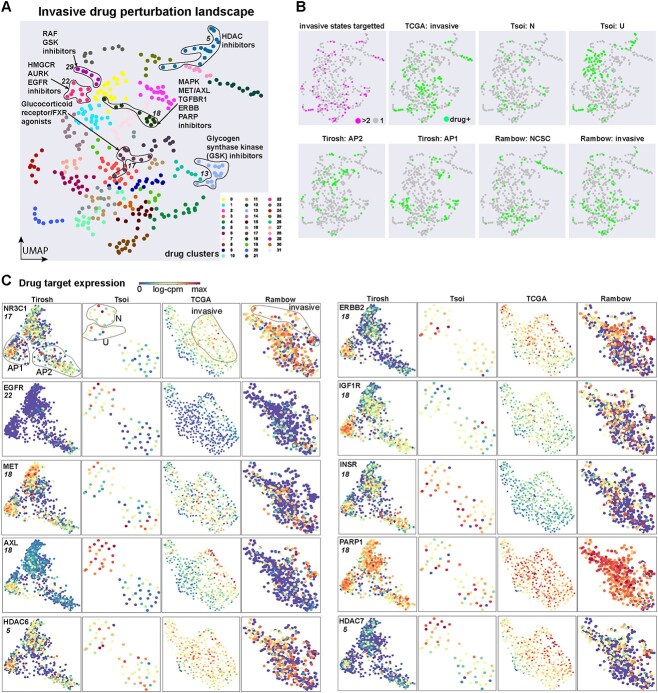
Cluster analysis of predicted invasive melanoma drugs reveals candidate drug classes for targeting invasive melanoma. (**A**) UMAP of LINCS1000 drug perturbation signatures, for drugs predicted to target invasive melanoma. Clusters of drugs with commonalities in MOA are highlighted. (**B**) The same UMAP as (A) repeated eight times with different drug meta data highlighted (top to bottom, left to right); drugs which target more than one melanoma invasive state (either in the same dataset or across independent analyses), drugs which target the TCGA invasive profiles, Tsoi neural-crest (N), Tsoi undifferentiated (U), Tirosh AP2, Tirosh AP1, Rambow NCSC and Rambow invasive. (**C**) For the highlighted drug clusters depicted in (A), the expression of the drug target genes are shown in each melanoma dataset. The drug cluster of the target is indicated below the gene name.

We developed an approach to identify mechanisms of action that are reproducible across studies, to circumvent potential false positives from individual drugs. For this analysis, only drugs that target any of the invasive states across studies are considered, since the presence of invasive melanoma is most highly correlated with poor patient outcomes [11]. To achieve this, DE gene lists between drug-treated and control cells are extracted from BeyondCell. A graph of drug-drug similarities is then constructed based on drug DE gene list overlap count is then used to cluster drugs and visualize their similarity using UMAP (Figure 7A; see Methods for full details).

Despite considerable variability in the drugs identified to target invasive states across studies (Figure 7B), we do find reproducible mechanisms of action when considering drug clusters. Drug cluster 5 corresponded to HDAC inhibitors (Figure 7A; Table S3). Cluster 13 to RAF and glycogen synthase kinase (GSK3B) inhibitors. Cluster 22 grouped HMGCR/AURK and EGFR inhibitors. Cluster 18 grouped MAPK, MET/AXL, TGFBR1, ERBB and PARP inhibitors. Cluster 13 grouped glycogen synthase kinase (GSK) inhibitors. While cluster 17 grouped agonists for the nuclear hormone receptors, including Glucocorticoid receptor (NR3C1) and FXR (NR1H4).

We then examined the expression of the genes encoding the proteins targeted by the drugs with common mechanisms of action. Consistent expression of these genes indicates that the drug target is consistently active in invasive melanoma regardless of study (Figure 7C). Several promising drug targets were inconsistently expressed, including EGFR, MET and ERBB2 (Figure 7C). Several drug targets were consistently expressed, including the glucocorticoid receptor NR3C1, AXL, PARP1 and several HDAC genes (Figure 7C). In line with these observations, survival analysis based upon the TCGA data showed a significant association of NR3C1, AXL and PARP1 expression (but not HDAC6/7), while no apparent association with survival for EGFR, MET and ERBB2 expression was observed (Supplementary Fig. 2).

## DISCUSSION

Phenotypic plasticity of melanoma is an important consideration for treatment since transcriptional subtypes exhibit distinct responses. A significant number of studies aiming to characterize melanoma phenotypic heterogeneity have been conducted with this rationale, primarily via transcriptional profiling. Here, we systematically compared landmark studies examining melanoma heterogeneity conducted using bulk and single-cell RNA-seq, derived from different melanoma models and in the context of drug treatment and adaptation. Importantly, we find an earlier study by Tsoi *et al*. using bulk RNA-seq masked broader melanoma heterogeneity, with some of the bulk RNA-seq based melanoma states representing an average of distinct melanoma states when compared with scRNA-seq data.

The Tirosh and Rambow scRNA-seq studies identified equivalent melanoma states, but several genes important for melanoma biology exhibited differential co-expression. Variabilities in the source context, such as drug treatment of tumour samples likely contributed to the observed co-expression differences. Tirosh directly sampled cells from human patient tumours, and Rambow sampled cells from clonal melanoma cells that were seeded into mice and treated with RAF/MEK inhibitors. Therefore, it is plausible that differences in the tumour microenvironment between host species or drug treatment underpinned altered melanoma gene expression patterns.

To understand the therapeutic relevance of the observed differences in gene expression between studies, we compared predicted drug sensitivities. We found that few drugs consistently targeted cell states between studies, but several plausible mechanisms of action were consistent among the predicted drugs. The most consistent candidate drug treatments for invasive melanoma were glucocorticoid receptor (NR3C1) agonists, AXL inhibitors, PARP1 inhibitors and HDAC inhibitors, as target gene expression for drug families predicted to target invasive melanoma. This suggests that invasive melanoma may be particularly susceptible to these drug classes. Previous studies have found glucocorticoid receptor agonism [32], AXL inhibition [33] and HDAC inhibition [34] can overcome treatment resistance in melanoma. PARP1 has been previously associated with aggressive melanoma [35], but has shown context-dependent efficacy on melanoma cell death and invasion [36]. Hence PARP1 inhibition for overcoming invasive/resistant melanoma represents a novel prediction that could explain the previously observed context-dependent effects. Several drug classes under active investigation showed inconsistent target gene expression, including EGFR inhibitors [37], MET inhibitors [38] and ERBB2 inhibitors [38].

Interestingly, MET expression was consistently absent from all invasive-like states identified in the Rambow single-cell data, which differed from the other studies by the aforementioned use of seeding melanoma cells in a mouse model and treating with RAF/MEK inhibitors. Interestingly, several differences in gene co-expression between the Tirosh and Rambow studies were observed, and it is tempting to suggest that the down-regulation of MET in the Rambow invasive states may be in response to the drug treatment. Pierce, et al (2020) showed increased expression of MET regulated by transcription factor BRN2 increased melanoma resistance to extracellular matrix detachment mediated cell death (anoikis) [39]. Thus, MET inhibition induced cell death in non-adherent conditions. Furthermore, MET amplified non-small cell lung cancers have been shown sensitive to MEK inhibition [40]. The specific absence of MET expression in the Rambow invasive states suggests selection for non-MET expressing invasive cells under RAF/MEK inhibition, thus suggesting proliferative to invasive phenotype switching adapted to the drug-treatment utilized. This observation could not have been made without comparing melanoma invasive states across studies and under different conditions, suggesting cross-study comparisons of cell states could be used to identify potential drug-specific adaptive resistance mechanisms during melanoma phenotype-switching.

We identified several points of difference with the inferred relationships of the melanoma states compared with the review by Rambow and Goding (2019), and significant differences in gene co-expression between the Tirosh and Rambow single-cell studies. This underscores the importance of conducting data-driven cross-study examinations as done here. Such examinations can reveal key insights, including points of similarity and differences between studies in relation to their experimental design. In this study, we discovered masking of heterogeneity, differential gene co-expression, predicted drug sensitivities, and potential melanoma plasticity to confer resistance or adaptability to particular drugs. This is evidenced by cases of study-specific down-regulation of drug target genes shown here.

In this study, we unveiled data-driven relationships of melanoma heterogeneity between studies and used them to examine potential cross-study drug sensitivity. This allowed us to generate hypotheses regarding melanoma plasticity to evade specific drug classes. We suggest that future studies investigating melanoma phenotype-switching must recognize that bulk-based assays may skew the perceived melanoma states created. To reveal potential drug-specific adaptive resistance mechanisms, researchers using scRNA-seq in drug treatment conditions should compare drug-treatment trajectories with the trajectory obtained from Tirosh scRNA-seq data from human patient tumours. This will enable uncovering crucial insights into specific drug resistance mechanisms and improving melanoma treatment outcomes.

**Table 1:** Key resources table

**Dataset**	**Reference**	**Source**
Human patient scRNA-seq	Tirosh (2016) [9]	https://www.ncbi.nlm.nih.gov/geo/query/acc.cgi?acc=GSE72056
Low-passage cell line bulk RNA-seq	Tsoi (2018) [8]	https://www.ncbi.nlm.nih.gov/geo/query/acc.cgi?acc=GSE80829
Human patient bulk RNA-seq (TCGA)	Akbani (2015) [13]	https://portal.gdc.cancer.gov/projects/TCGA-SKCM
Human melanoma from mouse scRNA-seq	Rambow (2018) [7]	https://www.ncbi.nlm.nih.gov/geo/query/acc.cgi?acc=GSE116237
melanoma functional gene sets	Faletta (2017) [20]	https://genesdev.cshlp.org/content/suppl/2017/01/17/gad.290940.116.DC1/Supplemental_Table_4.xls
**Software**	**Reference**	**Source**
Monocle (v2.10.1)	Qiu (2017) [17]	https://bioconductor.org/packages/release/bioc/html/monocle.html
fastx_trimmer (v0.0.14)	Hannon (2010) [41]	http://hannonlab.cshl.edu/fastx_toolkit/
RSEM (v1.2.25)	Li (2011) [57]	https://github.com/deweylab/RSEM
amap	Lucas (2019) [43]	https://CRAN.R-project.org/package=amap
sklearn (v0.21.3)	Pedregosa (2011) [44]	https://scikit-learn.org/stable/install.html
GSEA	Subramanian (2011) [21]	https://www.gsea-msigdb.org/gsea/index.jsp
TRIAGE	Shim (2020) [45]	https://github.com/woojunshim/TRIAGE
gseapy (v0.9.16)	Chen (2013) [46]	https://gseapy.readthedocs.io/en/latest/introduction.html
EPIC (v1.1.532)	Racle (2017) [48]	https://github.com/GfellerLab/EPIC
Scanpy (v1.9.1)	Wolf (2018) [49]	https://scanpy.readthedocs.io/en/stable/
EnsDB.Hsapiens.v79	Rainer (2017) [58]	https://bioconductor.org/packages/release/data/annotation/html/EnsDb.Hsapiens.v79.html
Scanorama	Hie (2019) [51]	https://github.com/brianhie/scanorama
BBKNN	Polański (2020) [52]	https://github.com/Teichlab/bbknn
Dcanr	Bhuva (2019) [54]	https://www.bioconductor.org/packages/release/bioc/html/dcanr.html
Cytoscape	Shannon (2003)	https://cytoscape.org/
BeyondCell	Fustero-Torre (2021) [16]	https://github.com/cnio-bu/beyondcell
GEPIA2	Tang (2019) [56]	http://gepia2.cancer-pku.cn/

### Monocle trajectory inference

Pre-processed scRNA-seq count matrices and annotations produced by Tirosh *et al*. (2016) were downloaded from the Gene Expression Omnibus (GEO) under accession ID GSE72056 [9]. DE genes (Bonferroni corrected *P* < 0.05) were determined using a one-versus-rest approach with two-tailed *t*-tests comparing the reproduced bulk RNA-seq melanoma states from Tsoi *et al*. (2018) (GEO GSE80829 [8]). These DE genes were used for trajectory inference on the malignant Tirosh melanoma cells with Monocle v2.10.1 [17].

### Tsoi bulk RNA-seq preprocessing

Raw fastq bulk RNA-seq data was downloaded from GEO at accession GSE80829. The first 13 base pairs of the paired-end reads were removed using fastx_trimmer v0.0.14 [41]. Read counts per gene per bulk sample were generated using RSEM v1.2.25 against the Hg19 reference genome with settings rsem-calculate-expression –paired-end –bowtie-path bowtie-1.2.2-linux-x86_64 –bowtie-n 1 –bowtie-m 15 –bowtie-e 99999999 –estimate-rspd. Transcript per million (TPM) normalised gene expression was transformed to log2(TPM+1).

### PCA trajectory inference

A non-parametric, within-cell approach for quantifying gene expression profiles was utilised to remove batch effects in the Tirosh scRNA-seq data prior to PCA [42]. Gene expression profiles were converted into a binary representation of relative expression between gene pairs.

Let $G_s$ and $G_b$ represent the set of genes measured in the Tirosh scRNA-seq and Tsoi bulk RNA-seq, respectively. S represents the Tirosh gene expression matrix, such that $S_{ij}$ represents the expression of gene $j$ in cell $i$. B represents the Tsoi gene expression matrix preprocessed as described above. Gene pairs used were thus determined by 


(1) \begin{align*} & G_1 = G_s \cap G_b \end{align*}

(2) \begin{align*} & I_1 = \{ j\ |\ G_s[j]\ \epsilon\ G_1 \wedge\ 0 \le j < \left| G_s \right| \} \end{align*}

(3) \begin{align*} & M = \{median(\left| S_{:j} - median(S_{:j}) \right|)\ |\ j\ \epsilon\ I_1) \end{align*}

(4) \begin{align*} & I_2=argsort(M)[-3000:] \end{align*}

(5) \begin{align*} & I_3=\{I_1[j]\ |\ j\ \epsilon\ I_2\} \end{align*}

(6) \begin{align*} & G_2=\{G_1[j]\ |\ j\ \epsilon\ I_3\} \end{align*}

(7) \begin{align*} & P_1=\{\{n, m\}\ |\ n\ \epsilon\ G_2\ \wedge\ m\ \epsilon\ G_2\} \end{align*}

(8) \begin{align*} & R_{:p}=\{\textbf{1}_{B_{:P_1[p][0]}>B_{:P_1[p][1]}}\ |\ 0 \le p < \left| P_1 \right|\} \end{align*}

(9) \begin{align*} & C=\{\frac{\sum_{0}^{rows(R)} R_{:p}}{rows(R)}\ |\ 0 \le p < \left| P_1 \right| \} \end{align*}

(10) \begin{align*} & P_2=\{P_1[p]\ |\ 0 \le p < \left| P_1 \right|\ \wedge\ 0.49 \le C[p] \le 0.51\} \end{align*}

(11) \begin{align*} & T_{:p}=\{\textbf{1}_{ S_{:P_2[p][0]}>S_{:P_2[p][1]}} \ |\ 0 \le p < \left| P_2 \right|\} \end{align*}

where $P_2$ is the final set of gene pairs (Table S1), and $T$ is the relative expression matrix for the Tirosh data such that $T_{ip}$ is the relative expression of gene pair $P_2[p]$ in cell $i$. The Tirosh cells were then clustered using complete linkage hierarchical clustering with Euclidean distance using the amap package [43]. PCA was then performed for visualization of the single-cell relative expression profiles using sklearn v0.21.3 [44](Table S1). DE genes (Bonferroni-corrected *p* < 0.05) were determined one-versus-rest comparison of clusters with Wilcoxon Rank-Sum tests (Table S1).

### Gene set enrichment analysis

Spearman’s rank order correlation between the expression of each gene across cells and the cell PC values. For each of PC 1 and 2, significantly correlated genes (Bonferroni-corrected *p* < 0.05) were ranked from most positively correlated to most negatively correlated with the respective PC. GSEA was then performed against the C5 GO biological process Molecular Signature Database [10, 22].

### Melanoma gene enrichment

TRIAGE [45] was used to select 500 upregulated genes from the Tirosh cluster DE gene lists that had the highest discordance score by multiplying the repressive tendency score by the Wilcoxon Rank-Sum location parameter from performing one-versus-rest cluster comparisons (Table S2). The Enrichr method [46], as implemented in gseapy v0.9.16, was used to test for enrichment of these upregulated genes in melanoma gene sets compiled by Falletta *et al*. (2017).

### Transforming other datasets into the same PCA space

Relative expression using the same gene pairs (P2, Table S1) were calculated on the Tsoi and TCGA normalised expression matrices. These were then transformed into the same PCA space by taking the vector dot-product of the gene pair weights for each PC (determined from the Tirosh relative expression matrix) and the Tsoi/TCGA relative expression matrices using Sklearn [44].

### Selecting marker genes

Genes which were specifically expressed in each Tirosh cluster were taken as the top 80 with the highest fold-change in expression that were within the lowest scoring group of genes determined using sklearn.cluster.KMeans (n_clusters=10) on the specificity distance (s) (Table S2). Letting S[c],j denote the expression of gene j across all cells of Tirosh cell state c, such that c represent melanoma states M, T, AP1, or AP2, the specificity distance is defined as in equation 12: 


(12) \begin{align*}& sd_{ij} = 1-\frac{u_j\bullet v_c}{\left\| u_j \right\|\left\| v_c \right\|}\end{align*}

where 


(13) \begin{align*} & u_j = \{\frac{\sum_{}^{} \textbf{1}_{S_{[k],j}}>0}{\left| S_{[k],j} \right|}\ |\ k\ \epsilon\ \{M, T, AP1, AP2\} \} \end{align*}

(14) \begin{align*} & v_c = \{ \textbf{1}_{c=k}\ |\ k\ \epsilon\ \{M, T, AP1, AP2\} \} \end{align*}

The specificity distance is constrained between 0 (for perfect specificity) and 1 (perfectly not expressed in the cluster).

**Figure 8:**
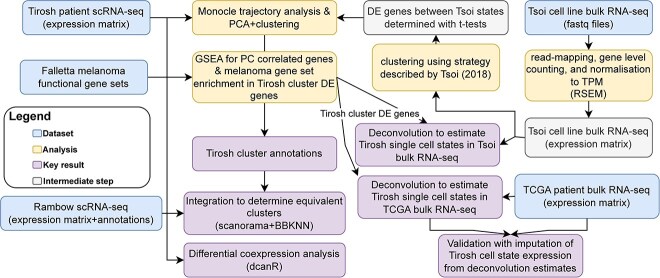
Flowchart of datasets, analysis performed, and key results.

### Deconvolution of Tsoi trajectory

Proportions of the Tirosh melanoma states within each of the Tsoi bulk samples were determined using a python implementation of the CIBERSORT method [47]. Average gene expression for each marker gene was determined in the Tirosh single-cell clusters (x). Nu-Support Vector Regression was then performed with the expression of the marker genes in each Tsoi bulk sample as the response variable (y) and the cell-type-specific expression of each gene as the predictor variables (see equation 15): 


(15) \begin{align*}& y\sim \beta x\end{align*}

Regression was repeated with different values of the Nu parameter - 0.25, 0.5 and 0.75. The Nu value that yielded the smallest mean square error between the predicted bulk gene expression from the regression ($y=\beta x$) and the actual bulk gene expression ($y$) was taken as the best fitting regression. The coefficients ($\beta $) from this regression were normalised to sum to one to estimate the cell state proportions within each bulk sample.

### TCGA analysis

We first downloaded bulk RNA-seq count matrices of TCGA samples corresponding to the SKCM project and normalised the data to TPM. We then selected markers genes that differentiate the melanoma states as well as immune cells using the method described above (Table S2). The Tirosh gene expression of these marker genes was then converted into TPM by reversing the log-transform and used to deconvolute the TCGA samples with EPIC v1.1.532 [48].

Imputed Tirosh cell state marker gene expression was determined from the TCGA bulk samples using a similar method described by Newman *et al*. (2019) [23]. For each gene ($i$), the cell-type specific expression of that gene ($i$) is estimated by regressing the expression of that gene in multiple bulk samples ($y_i$) as a function of the estimated proportion of cell states across the bulk samples (p, see equation 16): 


(16) \begin{align*}& \bar{p_i} = \frac{\sum_{j=0}^{n} p_{ij}}{n}\end{align*}

This was performed for each marker gene independently by using Lasso regression, as implemented in sklearn with parameters ‘alpha=0.0001, fit_intercept=False, and positive=True’.

Subsequent to the TCGA deconvolution, we then log2 transform the TPM expression values, prior to running the standard scanpy workflow for single-cell analysis [49]. Highly variable gene selection, PCA, sample-sample neighbourhood graph construction, and UMAP were all determined using Scanpy default parameters; with the exception of min_mean=1, max_mean=11 and min_disp=1 for highly variable gene selection. Leiden clustering was performed with resolution 1.5 followed by cluster marker genes determined by *t*-statistic ranking in Scanpy of genes comparing each cluster versus all other samples. Based upon the top marker genes, clusters were manually merged if no clear gene expression differences were evident, and labelled based upon the expression of the marker genes with known correspondence to skin, melanoma and immune content. Merging of clusters without evidence of distinct marker gene expression is inspired by a recent statistical approach for cluster significance analysis [50].

### Rambow single-cell RNA-seq analysis

Preprocessed read counts were downloaded from GEO at accession GSE116237 [7]. Cell annotations were provided by the original authors. Ensembl IDs were converted into gene names using the EnsDB.Hsapiens.v79 R library. Genes found in less than 10 cells were remove followed by normalization and log transformation using Scanpy. The top 2000 highly variable genes were then selected prior to performing PCA using Scanpy.

Highly variable genes were selected on the concatenated expression matrix of the Tirosh and Rambow data using Scanpy and batch_key=’dataset’ as input to scanpy.pp.highly_variable_genes(). Both datasets were then subsetted to these highly variable genes and z-scaled per gene within each dataset. Scanorama was then applied with default parameters to derive a 100-dimensional batch corrected single value decomposition (SVD) space [51]. Balanced Batched K-nearest Neighbours (BBKNN) [52] was then applied on the corrected SVD space to construct the cell-cell neighbourhood graph prior to UMAP embedding with Scanpy default parameters. Leiden clustering was then performed on the cell-cell graph with resolution 0.9 to obtain the joint clusters [53].

To determine differential gene co-expression between the Tirosh and Rambow scRNA-seq datasets, we utilised Dcanr [54] with the union of the top 2500 highly variable genes from each of the datasets determined with Scanpy. *Z*-score and spearman correlations were determined using dcScore() with dc.method=zscore’ and cor.method=spearman’ as input parameters. DcTest(), DcAdjust() and dcNetwork() were then run to calculate *P*-values, perform false discovery rate correction (FDR), and generate significant gene-gene correlations between the two datasets (FDR-corrected *P* < 0.01). Cytoscape visualised the differential gene co-expression for key melanoma genes [55].

### Drug susceptibility and survival analysis

Drug susceptibility predictions for cell states within each study were determined using BeyondCell [16]. DE gene lists for drugs predicted to target at least one invasive state across studies were then extracted from BeyondCell. Drug DE gene lists in these profiles were used to construct matrix D, such that for drug *d* and gene *j* each element in D is calculated as in equation (17): 


(17) \begin{align*}& D_{d,j}=\begin{cases} 1, & j\ \varepsilon\ G_{d,up}.\\ -1, & j\ \varepsilon\ G_{d,down}.\\ 0, & \text{otherwise}.\end{cases}\end{align*}

Where $G_{up}$ and $G_{down}$ is the set of up- and down-regulated genes by drug d, respectively. D was then used to construct a drug signature nearest-neighbor graph with scanpy.pp.neighbors() and metric=’hamming’. Drug signatures were then clustered using the Leiden method (resolution=2.95) and visualised using UMAP.

Survival analysis based on the identified drug-target genes we performed using GEPIA2 [56] with default settings, except 'Quantile’ was set as the method for the 'Group Cutoff’ parameter.

**Key Points**

- Melanoma progression involves changes in transcription and epigenetics that coordinate switching between a proliferative to an invasive cell state (termed 'phenotype-switching’).
- Microarray, bulk and single-cell RNA-sequencing have been utilised to develop data-driven models of gene expression changes during phenotype switching.
- By comparing these studies, we identified that bulk-based studies distort gene co-expression and obscure transcriptionally distinct melanoma cell states. Additionally, we described differential co-expression of genes between single-cell studies.
- Inconsistencies of gene expression between studies were observed, including expression of drug-target genes such as EGFR, MET, and ERBB2 within invasive melanoma. Other genes such as NR3C1, AXL, PARP1 and several HDAC genes were consistently found in invasive melanoma.

## Supplementary Material

FigS1_TRIAGE_500_genes_2_elad055

FigS2_SurvivalAnalysis_elad055

Table_S1_trajectoryDerivation_elad055

Table_S2_markers_elad055

Table_S3_beyondcell_results_elad055

## Data Availability

Manuscript code has been deposited at https://github.com/BradBalderson/Melanoma_manuscript. All datasets, which were analysed as shown by Figure 8, are publicly available and linked in the Key Resources Table.
