# Supplementary figures and images for: Systematic analysis of the transcriptional landscape of melanoma reveals drug-target expression plasticity
Source: Brief Funct Genomics. 2024 Jan 5;24:elad055. doi: 10.1093/bfgp/elad055 (PMC11979751; doi:10.1093/bfgp/elad055)

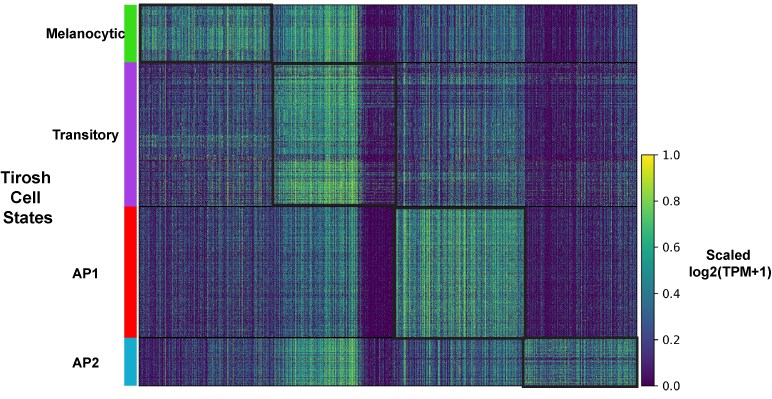

Supplement: FigS1_TRIAGE_500_genes_2_elad055 [file figs1_triage_500_genes_2_elad055.jpeg]

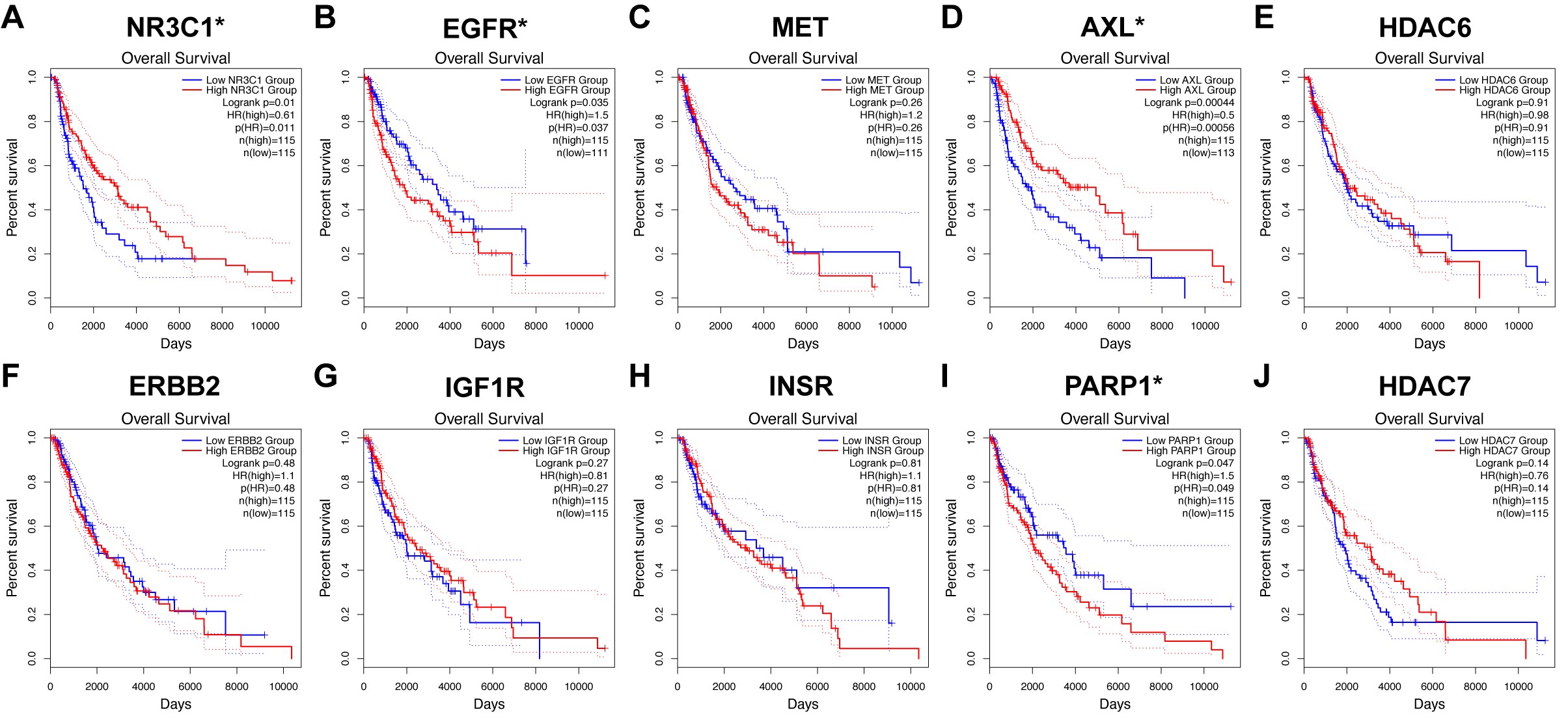

Supplement: FigS2_SurvivalAnalysis_elad055 [file figs2_survivalanalysis_elad055.jpeg]
